# The intended and unintended consequences of remote working: Narratives from a sample of female public service managers in South Africa

**DOI:** 10.3389/fpsyg.2022.949914

**Published:** 2022-10-13

**Authors:** Willie Tafadzwa Chinyamurindi

**Affiliations:** Department of Business Management, University of Fort Hare, Alice, South Africa

**Keywords:** managers, COVID-19, female, remote working, South Africa

## Abstract

The COVID-19 pandemic has affected the world of work. Stemming from this, new forms of work arrangements are proposed. One such arrangement concerns the use of remote working. Scholars appeal for more empirical inquiry into such work arrangements as an unintended consequence of the COVID-19 pandemic. The study narrows its focus to investigating remote working experiences from the lens of female middle managers operating within the South African public service. A qualitative research approach utilizing narrative inquiry of 23 female middle managers was used. Based on the analyzed data, remote working is illustrated from the participant experience as having intended and unintended consequences. In illustrating these dual consequences is a nexus between opportunities and challenges. Based on the identified intended and unintended consequences as findings, interventions have been proposed that impact not just the experience of being a middle manager in the public service but also strategies in dealing with remote working. At the core are strategies for individuals and organizations. These strategies potentially allow for middle manager contributions to be enhanced while also enhancing organizational outputs while working from home.

## Introduction

The COVID-19 pandemic has affected society. The world of work has not been spared from the impact of the pandemic (Hooley et al., 2020). In developing countries such as South Africa, proposals have been made for the need for interventions that are not only reactive but also proactive in response to the pandemic (Dowdeswell and Kriek, 2021). Remote working has emerged popular and preferable as a response to the COVID-19 pandemic (Taser et al., 2022). Within remote working, employees have the opportunity to work from home or away from the office enabled by technology (Molino et al., 2020). This allows for the pursuit of not just individual but also organizational goals, albeit challenges such as those created by the COVID-19 pandemic (Potgieter et al., 2021).

From the literature, calls exist in understanding the impact of remote working as a work arrangement stemming from the COVID-19 pandemic (Motamarri et al., 2022). For instance, how managers exercise managerial responsibilities within a remote working setting is argued as needing further inquiry (Henry et al., 2021). Equally important, how employees respond to such managerial influence is deemed key in informing understanding around aspects related to remote working (Potgieter et al., 2021). The focus here is on understanding the range of individual and organizational resourcing tactics and how they manifest in making remote working a success (Koekemoer et al., 2021). In seeking such an understanding, interventions can be proposed around ensuring individual and organizational resilience capabilities are created stemming from the disruptions of the pandemic through remote working (Maruping et al., 2021).

The world of work is noted to be changing due to the impact of the COVID-19 pandemic (Hossain, 2020). The functionary tasked to deal with people issues must be seen to be responsive to these changes (de Klerk et al., 2021). For the consideration of remote working to be incorporated, the human resource component must align the individual and organizational ideals together (Donnelly and Johns, 2021). In achieving this, the needs of employees must be considered (Chinyamurindi et al., 2021). Such alignment becomes crucial and places into focus the need for a competency-based framing to be in place (Cooke et al., 2020).

In allowing for flexible work arrangements, a need exists for organizational architecture that supports this (Williams et al., 2021). There is a noted scant focus in studies that investigate remote working in response to the COVID-19 pandemic (Jamsen et al., 2022). Furthermore, how remote working influences managerial actions, including the gender aspect, remains an angle not explored within the literature. Managers are deemed crucial as response agents for the organizational interventions to succeed (Ahlqvist et al., 2020). Based on this, this study aimed at exploring the remote working experiences of a sample of female middle managers in the South African public service. The following research question was set:

What are the consequences of remote working from a sample of female middle managers working in the South African context?

Informed by the quest to answer the proposed research question, the next section details a literature review for the study consisting of the theoretical and empirical literature.

## Literature review

### Theoretical lens

In setting the theoretical position of the study, there is a need to consider some views from the literature. First, there appears to be no one theoretical lens that can be useful in understanding aspects related to remote working (Matli, 2020). This also extends to the very definition of what remote working is (Sullivan, 2003). This could be due to the multiple interpretations that can be deduced with regard to the concept of remote working. At the core, though, should be the view that remote working serves the ideal of assisting in developing human capability (Doern, 2016). The ultimate aim is the attainment of competitive advantage (Bag and Gupta, 2019). Technology in varying forms allows for such attainment (Spicer et al., 2021). This has been noted to be important, especially given the challenges stemming from the COVID-19 pandemic (Mariani and Nambisan, 2021; Mariani et al., 2021).

In developing a theoretical lens for this study, social exchange theory is considered (Homans, 1958). The underlying premise here is that human behavior is an exchange between goods and services within a social context. In essence, the exchange between these goods and services results in behaviors by individuals (Blau, 1964) and is a source of motivation and attitudes (Felstead and Henseke, 2017). Through remote working, a set of intended and unintended consequences can emerge (Choi, 2018). Through such exchange, some demands and resourcing behaviors can potentially emerge, as also espoused in the job demands-resources model (JD-R; Bakker and Demerouti, 2007, 2017). The combination of social exchange theory and the JD-R provides a useful framework that tries to explain the role that working conditions may have on individual psychological wellbeing.

When individuals perceive a work situation and its demands to be high and support services limited, this may result in negative experiences. Noted experiences here include burnout, emotional exhaustion, depersonalization, and a reduction in professional efficiency (Bakker and Demerouti, 2007; Bakker et al., 2011). Given that remote working exists as a new form of work in response to the COVID-19 pandemic, this may elicit responses from individuals. These responses may either be positive or negative, depending on the work situation or support services in place.

### Empirical literature

The literature affirms some benefits and challenges around remote working.

Concerning the benefits of remote working, these are acknowledged to impact individuals and organizations in general. Chiefly, remote working is praised for how work and private life can be integrated together (Allen et al., 2015). Such flexibility has the potential to promote work-life balance and also the wellbeing of the employee and the organization (Wang and Haggerty, 2011).

Potentially such flexibility between the home and the workplace may also reduce work-family conflict (Allen et al., 2013). Others apportion an added benefit to an environmental gain. Working from home has been noted to reduce the time spent in traffic and potentially reduce carbon emissions (Sutton-Parker, 2021).

In allowing for remote working, the technology interface is noted to be important and crucial for success (Blagoev et al., 2018). The desire here is for technology to be in place that supports remote working and reduces any anxiety associated with this. Technological investments may need to be in place in the form of hardware and software that allow for home and office support (Meijerink et al., 2018; Song et al., 2020). This must also be supported by an organizational culture that supports remote working ideals (Donnelly and Johns, 2021). In essence, an investment is needed not only financially but also through support structures, including policies for remote working to be a success (Kornberger et al., 2017). Much of the failures around the remote working stem from technological failure and hence the need to address and put in place support services for this (Karl et al., 2021).

In developing countries like Ghana, the interacting role of factors such as technology, organizational design, and environmental characteristics has been found to affect the adoption of remote work systems (Ofusu-Ampong and Acheampong, 2022). In essence, remote working in tandem with organizational support efforts offers some flexibility for employees (Chatterjee et al., 2022).

In some contexts, employee support was needed in adjusting to the new normal way of work. Estrella (2022) noted a lack of preparedness toward remote working as a contributor to its failure in some organizations. This was heightened given the uncertainty created by the COVID-19 pandemic. This lack of preparedness and support structures in place has led to stressful conditions, especially for employees (Cristea and Leonardi, 2019). Others note remote working challenges from an organizational perspective as leading to exhaustion and permanent feelings of disengagement (Walker et al., 2022) including also job insecurity concerns (Ghislieri et al., 2022).

In allowing for employees to work from home, some precursors need to exist. Some place caution on the challenge that comes with the excessive use of technology as potentially leading to technostress and loneliness (Taser et al., 2022). In the long run, these negative psychological effects may have far-reaching consequences on the performance not just of the individual but also of the organization (Charalampous et al., 2019). In curbing this, remote working should be supported by digital resilience and sensitivity awareness in assisting all employees (Tramontano et al., 2021).

The existence of supervisor support is needed and is coupled with a work culture that allows for this (Hoch and Kozlowski, 2014). Internal mechanisms of support need to be in place to assist a remote work learning culture (Blagoev et al., 2018). This can include the necessity for continuous employee engagement and organizational buy-in (Syed, 2020). Management support becomes crucial in the support of a remote working environment (Christianson and Barton, 2021). The role here is to use the management capability in implementing policies and practices that allow for remote working.

Another angle advocated for is the need to pay attention to the individual worker as an important conduit in allowing for the new work arrangements to emerge. Some ideas here include the necessity to understand the ensuing complexity that accompanies remote working (Mayer et al., 2021). Remote working has been attributed to lead to professional and social isolation (Carillo et al., 2021) and an increase in psychological distress (Saura et al., 2022; Van Zoonen and Sivunen, 2022). From here, support mechanisms can be provided to assist employees to adjust, especially with remote working arrangements (Mahadevan and Schmitz, 2020).

This study narrowed its focus on understanding remote working experiences from a gendered lens by paying attention to women working within the public service. Furthermore, a noted area of inquiry is how those in managerial responsibilities experience aspects such as remote working (Chinyamurindi et al., 2022). This is an aspect that has not received attention, especially from a developing nation context. Potentially, such an inquiry can assist in advancing understanding of the experience of female managers, especially within a public service deemed important for service delivery outcomes (Mahlasela and Chinyamurindi, 2020).

## Methodology

In answering the research question, the study incorporated a qualitative research approach, relying on the use of interviews with a sample of female public service managers. Such an approach and technique assisted in understanding not only human experience but also the ensuing complexity of this (Bryman et al., 2018). Furthermore, the usage of such an approach and technique assists in understanding human experience and sense-making processes individuals make in their context (Chinyamurindi et al., 2021). A list of questions asked is found in Appendix 1.

### Sample and data collection

A convenience sampling technique was used. Participants were recruited from provincial government departments in the Eastern Cape of the Province of South Africa. For the purpose of the study, a sample of female middle managers working at these provincial offices took part in the study. Letters of invite were sent to provincial heads of department, detailing the purpose of the study. From this, the heads of department were to circulate the correspondence to all members of their units who fit the criteria. A total of 23 female middle managers were recruited for the study. Table 1 presents the demographic profiles of the participating female managers. The following inclusion and exclusion criteria were used to select participants: a participant had to be (1) a middle manager who had been in their position for at least 2 years and (2) a female. The characteristics of the participants are presented in Table 1, notably the middle manager position that is occupied.

**Table 1:** Demographic characteristics of participants.

**Participant**	**Race**	**Years in middle manager position**	**Middle manager position**
1	Black African	7	Human resources
2	Colored	6	Supply chain
3	Black African	4	Accounting
4	White	3	Information management
5	Colored	6	Supply chain
6	Black African	4	Legal
7	Colored	3	Human resources
8	Indian	6	Accounting
9	Colored	3	Information management
10	Black African	2	Human resources
11	Colored	6	Supply chain
12	Black African	7	Procurement
13	Colored	5	Human resources
14	Black African	3	Information management
15	Black African	7	Legal
16	White	4	Human resources
17	Black African	6	Accounting
18	Black African	4	Supply chain
19	White	7	Human resources
20	Black African	8	Procurement
21	Colored	10	Supply chain
22	Black African	4	Information management
23	Colored	6	Legal

### Ethical statement

Ethical guidance was adhered to as part of the study. First, ethical clearance was applied for before the research took place at the participating university. Second, and related to the first point, attention was given to the guidance of the Helsinki Declaration of 1972, which stipulates that when research is being conducted and the participants include humans or animals, the researcher has to get a clearance from the ethics committee (Parsa-Parsi, 2017). The researcher complied with regulations stipulated that include informed consent, right of participation, and confidentiality and anonymity of the research participants. Third, the researcher sought to avoid prejudice and ensure all participants enjoyed equal rights and participation in this critical project. Pseudonyms were used to avoid identifying the participating individuals, including the public service entities they work for.

### Strategies to ensure data integrity

Strategies were used to ensure data integrity. First, an interview schedule was developed, guided by the literature-informing questions asked to participants. This assisted in providing a theoretical foundation on which the study was based (Creswell, 2014). Second, in ensuring integrity, the formulated interview guide was also vetted by a panel of qualitative experts who assisted in improving aspects related to the questions to be asked to the participants. Such an expert pool assisted greatly in leading to the interview questions (Denscombe, 2003). Third, transcriptions were sent back to participants after the interviews (Wolcott, 1990); any changes were made as per the wishes of the participants. Finally, as proposed by Wolcott (1990), in undertaking data analysis, our goal was to bring to light issues we regard as critical in order to arrive at an informed interpretation of the findings.

### Data analysis

The study adopted a narrative analysis technique relying on the three levels of meaning-making (McCormack, 2000). Narrative inquiry assists in understanding the lived experiences of individuals based on the experiences they are or have gone through (Gatenby and Humphries, 2000; Brown, 2012). The three levels help assist in deducing meaning from a large set of data (Toolis and Hammack, 2015). The first of these levels assists in creating a vignette of each interview transcript, with the potential of this being developed into a longer narrative about each participant. Second, the developed narrative participant experiences are then compared to those of each participant that took part in the study. In the final stage, an elaboration of the developed narratives with the aid of quotations and experiences shared by the participants is then provided (McCormack, 2000). Narrative inquiry has been used previously in organizational behavior research and lauded for its effort in understanding human behavior and experience (Chinyamurindi et al., 2021; Mpetile and Chinyamurindi, 2021; Harry and Chinyamurindi, 2022).

## Results

Based on the data analysis, remote working was found to have intended and unintended consequences. Intended consequences consisted of those expectations that the female managers attributed to being part of the effect of remote working. These consisted of positives such as remote working (a) as a safety measure from the spread of the COVID-19 pandemic; (b) allowing for work-life balance; and (c) allowing for time to spend time with children and partners. However, remote working had some unintended consequences. These effects were mostly the emergence of issues not expected by the female middle managers as part of the experience of working from home. These also consisted mostly of negatives such as (a) technology failure; (b) people management challenges, especially with senior managers and subordinates; (c) extension of working hours, resulting in the blurring of work boundaries. These findings about the intended and unintended consequences of remote working are discussed next.

### Intended consequences

The first identified intended consequence was remote working as a safety measure from the spread of the COVID-19 pandemic. Participants appreciated the value of working from home as a safety net from the challenges posed by the pandemic. This appreciation came about based on the challenges the world and South Africa were facing as a result of the pandemic. This was a common narrative among participants. One participant expressed this succinctly:

“There was definitely benefit of working from home—key was that we were at the height of the pandemic. The biggest benefit—we were sheltered away from the spread of the pandemic. Especially given the open floor system we used—we were going to be at risk. Working from home allowed us a safety net.” **[Participant 19, Human Resources Manager]**.

Another participant expressed the remote working model as much needed especially given the personal challenges they had faced surviving the COVID-19 pandemic.

“I got COVID just at the start of the pandemic. I went through a hard long journey of recovery. With this was an appreciation of our health care system especially given the uncertainty of the pandemic. Given this experience, I just appreciated more the remote working model. We needed to work from home. Our health needed this.” **[Participant 7, Human Resources Manager]**.

Some participants continued to appreciate the value of remote working, especially as a response to the challenges stemming from the pandemic. In view of this, there was a clarion call from the participants for the need to rethink how we work given future pandemics.

“Remote working has indeed a future. We are probably going to experience future pandemics. We must be able to evolve with our work systems to the changes happening. Remote working definitely will limit the spread of any disease and protecting the health of employees. The COVID-19 pandemic experience was a useful learning period in preparation for the future.” **[Participant 1, Human Resources Manager]**.

The second identified intended consequence was remote working, which allows for work-life balance. Participants had general praise for remote working in allowing for work-life balance. One participant bemoaned the time they spent in traffic and how during the remote working experience use that time for exercise.

“Remote working helped restore the pact I had with my gym club and also a love for exercise especially when COVID-19 restrictions were being lifted. I would have had the opportunity to do all this unless I had been working at home.” **[Participant 6, Legal Manager]**.

For some, remote working allowed participants to be able to also balance their lives in view of expected societal roles.

“During the height of the pandemic, I could spend more time connecting with other facets of my life outside work. I could read more at home. Do some gardening. Even connect more with the world around me. Yes I got to do some work but I also managed to do things I could not do.” **[Participant 10, Human Resources Manager]**.

Remote working also allowed participants the opportunity to also take part in hobbies as a way of escape from the difficult conditions emanating from the pandemic. One participant appreciated this.

“Being at home allowed for convenience to also just escape from the work duties—do the school run and even attend extra mural activities for the kids. Yes, I would have my phone with me, but I appreciated being away from the desk and being able to manage other aspects of my life.” **[Participant 4, Information Management Manager]**.

Finally, remote working allowed time for the middle managers who took part in the study to spend time with their children and partners. One participant expressed appreciation for assisting their children with their homework while also doing their own while working remotely:

“I enjoyed remote working because it also fit in with my role as a care giver to my family. One of my children has special needs and working from home assisted me in spending more time with my kids. Yes I managed to get work done but I also managed to also monitor the development of my children especially during the height of pandemic with lockdown in place.” **[Participant 12, Procurement Management Manager]**.

To some participants, remote working also led to an appreciation of family values and also created time for the family structure. This became evident, especially since most of the family members were spending time at home. For some, remote working allowed participants in the study the opportunity to also be more present in the lives of their children.

“Home schooling was supported well with the remote working arrangement. I could easily monitor how the children were doing with their schoolwork while at the same I am doing my work.” **[Participant 8, Accounting Manager]**.

Finally, some participants appreciated the value of remote working as allowing them time to spend time with their partners.

“I think remote working also saved my marriage. My partner and I are appreciative of being in professions where we can work remotely. This assisted both of us to work in the same room at home. Such time was just the bond we needed. Remember in a week we usually spent half of the week at the office pre-pandemic. It was wonderful to work from home not just for the work aspect but also our relationship.” **[Participant 3, Accounting Manager]**.

Table 2 illustrates additional quotes supporting the first finding of the intended consequences of remote working. Based on Table 2, the participating managers using their functional areas illustrate the benefits of remote working.

**Table 2:** Intended consequences of remote working.

**Remote working as a safety measure from the spread of the COVID-19 pandemic**	**Remote working allowing for work-life balance**	**Remote working allowing for time to spend time with children and partners**.
“At the time of the pandemic and the lockdown, our surety was avoiding contact. Working remotely assisted us greatly.” **[Participant 23, Legal Manager]**.	“Being at home was an eye-opener. It allowed me a chance to have some form of control of my days in the early days. At the height of the pandemic, it became very difficult. There was uncertainty — we got tired of remote working — we missed the office spaces. The office had some of restriction. Working from home had convenience but there were no boundaries.” **[Participant 8, Accounting Manager]**.	“Remote working allowed us as parents to actually see our kids more. At the start of the lockdown it was tough — as we got used to the setting — it became better. Yes it was challenging but as parents we feel more involved in the lives of our kids as we work remotely.” **[Participant 12, Procurement Manager]**.
“Working from home has definite health benefits. For starters it allowed us not spreading the rate of infection. The working remotely model actually promotes better health.” **[Participant 5, Supply Chain Manager]**.	“I was able to attend to some other issues which I would not otherwise do. As a priority allowing for work-life balance. Something the day to day routine pre-pandemic would not allow.” **[Participant 20, Procurement Manager]**.	“Working remote allows me and my husband to have time for each other. We spend at least two days at home and add another two days of the weekend. So in a week we at least speak half of our time together. This assists our relationship given we just got married.” [**Participant 8, Accounting Manager]**.

Source: Based on the data analysis.

The second finding presents the unintended consequences of remote working. These consequences were negative and expressed concerns around remote working. These unintended consequences included (a) technology failure; (b) people management challenges, especially with senior managers and subordinates; and (c) extension of working hours, resulting in the blurring of work boundaries. These findings on the unintended consequences of remote working are discussed next.

### Unintended consequences

The first unintended consequence consisted of challenges related to the technology interface. A challenge here could be the abrupt nature of the transition to remote working. One participant attributed to a lack of readiness within the public service sector in general.

“We were not ready for remote working. The start of the process was really messy. All our computers needed to be loaded with software that allowed for remote working. Then we had worked around that challenge, the support service part was also an issue. What made this difficult was that our support services really never featured online but were stronger face to face. We were just not ready.” **[Participant 16, Human Resources Manager]**.

A second participant attributed this lack of readiness and technological failure to being caused by the inherent structural challenges within the public service.

“The entire public service system has its faults. Inherently the system was based on a face to face interface. Transitioning to technology would be a big challenge. When the technology was in place, we also had find ways to support especially our managers as we noticed more reports of technology failure. This placed need for us to invest more in training people in dealing especially with these technological challenges.” **[Participant 20, Procurement Manager]**.

Technology failure appears to be related to the challenges of old computer infrastructure that, in some cases, needed to be revamped. Furthermore, there was also a need for software support. Given the challenges of people working remotely, technology failure appeared inevitable.

“Challenges with remote working had much to do with technology failure. As a person in supply chain and I think given what we know, we need to be more savy especially for the future. Our Information Technology team attributed much of the challenges of remote working to technology issues.” **[Participant 21, Supply Chain Manager]**.

The second unintended consequence consisted of people management challenges, especially with senior managers and subordinates. This mostly involved challenges around working away from the office. One participant working in legal expressed as follows:

“When we started working remotely, I noticed some challenges especially working with both our managers and sub-ordinates. Most of the personnel had to submit their documents for legal opinions and vetting. I think our reliance on the face to face system really spoiled us. Documents were always late and not done correctly. This created strain. Worse all our interaction had to be done online.” **[Participant 23, Legal Manager]**.

Concerning the technology failure presented earlier, one of the study managers placed blame on a top management structure, not in tune with remote working.

“Most of the senior managers were not technologically savvy. The face to face interface at least for interaction. So now imagine the situation where you do not see each other. The middle managers had to do most of the work especially working remotely. You just don't know what is happening with our seniors. Most of which I think were merely managing over the phone. This does not work for me and even affects how we also relate with those below us.” **[Participant 20, Procurement Manager]**.

The middle managers also expressed concern with aspects related to working with senior managers who appeared to be using the remote working set to be absent from aspects of work. One participant expressed as follows:

“The remote working system if adopted well needs a working structure which breaks the traditional hierarchy system found within the public service. I think everyone is just curious if people are really working on the other side. This level of distrust brings a lot of questioning to a perfectly good model of working that I believe we should be adopting.” **[Participant 14, Information Management Manager]**.

A final unintended consequence of remote working puts into focus concerns around the extension of working hours, resulting in the blurring of work boundaries. Generally, participants generally felt that they were most accessible at any time for work-related issues. One participant expressed as follows:

“Working remotely has made me overly accessible. It simply means, your phone must be always open. In normal times you could just close the door or even use a secretary to stop being disturbed. This is not the case working from home.” **[Participant 3, Accounting Manager]**.

Another participant attributed this idea of being available at any time to be hidden in the idea of working from home but in a subtle use for surveillance. This view attributes concern to lie with top-tier management within the public service.

“I think with remote working we are deemed to be available due to some concerns that our senior managers have. This is no different to the big brother is watching mentality. We must be there to shield responses as is required.” **[Participant 15, Legal Manager]**.

Some participants then narrated concerns of a psychological nature, especially when deemed to be most accessible. This was expressed by one participant.

“Literally fatigued not with remote working but its unintended consequences. I cannot switch off my phone—I must sit in front of the desk at home all the time. This has led to stress for me. Even now when my phone rings, I am just shaking.” **[Participant 22, Information Management Manager]**.

Boundaries were blurred with remote working. The source of this could be attributed to the way in which reporting mechanisms work within the public service. Remote working, as expressed by one participant, showed these gaps.

“The organizational structure within the South Africa public service is still that one command and control. This work well within physical spaces. With working from home that command and control manifests in the excessive calls. Someone can call you late at night. That was salient nightmare for remote working for me.” **[Participant 17, Accounting Manager]**.

Table 3 presents additional quotes supporting the second finding of the unintended consequences of remote working. Based on Table 3, the participating managers using their functional areas illustrate their concerns with remote working.

**Table 3:** Unintended consequences of remote working.

**Technology failure**	**People management challenges**	**Extension of working hours resulting in blurring of work boundaries**
“Our supply chain processes were heavily dependent on paper-based working. So when submitting documents there is a reliance on signatures on the physical paper. Remote working revealed two things. The first thing was not sustainable. Second, remote working did not help me. Much of the responsibility in transitioning to online forms was the responsibility of my office. I was busy round the clock.” **[Participant 11, Supply Chain Manager]**.	“The pandemic gave us so much to think about as HR professionals. We were just not ready. Then we are told to work from home. One of the things that we needed to have (which we did not have) a policy around working from home. We had to pass this quickly—all this fell on me as the HR manager. So I need d to up my game. Sadly, it also meant attending to work issues at ridiculous hours at night.” **[Participant 7, Human Resources Manager]**.	“We were the interface of the organization as part of the ICT team. We got calls at any time of the day to assist especially those working from home. So yes I enjoying working from home but it seemed like we were still at the office.” **[Participant 14, Information Management Manager]**.
“Managing after-hours that's how I summarize remote working. You are just stretched to the limit. The limit is often framed by the one above you or below you. The early days of working from home where tough. After some time I mustered enough courage and would now set boundaries of what I can or cannot do. It was always clear I had to save my work schedule as I found myself going beyond the call of duty effort wise and also with my time.” **[Participant 20, Procurement Manager]**.	**“**Definitely observed more conflict working remotely. I think the face to face office structure allows people to see each other. This can create an impression of uniformity and that everyone is pulling together. Not having people at the office heightened some suspicion. What is really happening behind the computer at home was really a mystery and a source of apprehension.” **[Participant 19, Human Resources Manager]**.	“Remote working for some people implied a breaking away from the structure that comes with having appointments. Online meetings became painful – too many happening at the same time. The added challenge was that outside the meetings I had to have other meetings. This means working over-time and even meetings as late as 8 at night.” **[Participant 23, Legal Manager]**.

Source: Based on the data analysis.

## Conclusion and discussion

The aim of the study was to investigate the remote working experiences of female middle managers operating within the South African public service. The findings reveal the positives and negatives associated with remote working. These are expressed as intended and unintended consequences. These findings respond to how organizations and their members are responding to the COVID-19 pandemic. Through these findings of intended and unintended consequences, the impact of the pandemic on the world of work is illustrated. In essence, the study also shows how the public service in South Africa as a context of work has not been spared from the documented challenges of the pandemic (Hooley et al., 2020; Dowdeswell and Kriek, 2021). As a contribution, the study answers calls for studies that show such responses to the COVID-19 pandemic as a basis for enhancing understanding (Hossain, 2020).

In South Africa, as illustrated by this study, remote working appears to be a popular option, especially stemming from the challenges posed by the COVID-19 pandemic (Dowdeswell and Kriek, 2021). For the participating female middle managers, remote working appears to serve a utility that allows them to be able to achieve work-life balance. This finding confirms previous findings about the benefits of remote working (Molino et al., 2020; Potgieter et al., 2021). The call should therefore be for organizations that seek to align the work-life interface deemed important to workers in contemporary society (Donnelly and Johns, 2021). Flexibility here is crucial (Wang and Haggerty, 2011). The type of flexibility also allows for employees to be able to do those things they wish to do of their volition in terms of time and place. A convenience that remote working could offer (Sutton-Parker, 2021).

However, the participant findings reveal a caution around the blurring of boundaries, especially during office hours due to remote working. Based on the two findings, capabilities can be developed that assist employees, including managers, adjust to remote working (Maruping et al., 2021). The two findings proffer and assist understanding of not just the experience of remote working but also the needs to heighten focus toward an understanding of the needs of managers. Stemming from the COVID-19 pandemic, such an understanding assists in the roll-out of interventions to support employees within organizations (Chinyamurindi et al., 2021). As shown in this study, the needs and experience of managers for remote working are crucial as this leadership cohort is an important vehicle for change efforts within organizations (Ahlqvist et al., 2020).

Another interesting angle to the findings of the study stems from the sample base used as part of this study. The focus was on using female middle managers. The participants in the intended and unintended consequences of remote working place focus on priorities that are deemed important to their functioning based on their managerial tier. Furthermore, this appears to also inform responses to the COVID-19 pandemic (Motamarri et al., 2022). This study and its findings thus contribute to the call for research that also understands remote working from the managerial lens (Henry et al., 2021).

The finding around remote working resulting in extended working hours becomes crucial. The findings reveal the importance of the private lives of individuals (Allen et al., 2015). It becomes concerning when that private space is impeded in the name of work. In turn, as shown in previous research, when employees deem their personal spaces not respected, this may affect their wellbeing. This finding differs from previous research that mostly attributes remote working to fail due to technological failure (Karl et al., 2021). The challenge, as shown in this study, stems from people-related issues. An aspect here is the perception that working remotely makes one always available on technological devices. This was something the participating middle managers did not appreciate.

To the participating managers, remote working appeared to allow for convenience intentionally, but some challenges emerged. This seems consistent also with previous studies (Taser et al., 2022). From the findings, suggestions can be gleaned that can assist in improving aspects of remote working. Notably, the need to address technology and people issues around aspects of remote working (Molino et al., 2020). In addressing these issues, there is also a need to align individual and organizational goals to enable efficient processes of work (Potgieter et al., 2021).

## Implications

### Practical implications

The findings of the study can proffer useful practical implications, especially for functionaries such as the human resource management utility. Based on this, sound people management strategies can be proposed based on the body of evidence from this study (de Klerk et al., 2021). First, given the challenges presented with remote working, strategies can be drawn around this to assist female managers in dealing with remote working. For instance, the finding around the extended office is a cause of concern. The female managers participating in this study shared concerns on how working remotely merely extended their duties even beyond the hours of work. Managers must be assisted in exercising their managerial responsibilities, especially within a remote working setting (Henry et al., 2021). The need here is for organizational architecture that also supports remote working (Williams et al., 2021).

Practical steps can be drawn around assisting employees, especially in dealing with remote working. Second, there appears to be a positive support for remote working based on the narratives of the female managers. Organizations may need to start incorporating remote working proactively stemming from the revelations and experiences of the COVID-19 pandemic. Policies can be put in place inside the organization to support this new normal of working (Jamsen et al., 2022). Furthermore, employees may need to be capacitated through training interventions in them improving their technological competencies that assist with remote working. Finally, linking performance management systems to remote working can be useful in achieving the balance between the home and work interface. It would appear that remote working assists in allowing individuals to have control of their time. Such control may have a dual impact that benefits the home and work sphere. These suggested steps could fit in with individual and organizational resourcing tactics and how they can assist with remote working (Koekemoer et al., 2021).

### Limitations and future studies

Some limitations can be pointed out in this study. First, the research is not generalizable to the entire population of female middle managers working in the South African public service. The sample used assisted in generating understanding around remote working experiences. Therefore, caution is to be exercised when interpreting the findings. Future research could also draw on the comparison between male and female managers and improve on the sampling challenges from this study. Second, the study notes a limitation experienced, especially during the data collection process. The data were collected at the height of the pandemic. Future research may use longitudinal measures or multiple points of data collection with the sample. This could assist in addressing the challenge of collecting data at one single point in time. Future research could also incorporate quantitative research methods in testing determinants of remote working experiences through model testing. This potentially (like the qualitative method used here) assists in enhancing the understanding of remote working as a construct.

## Data availability statement

The datasets presented in this article are not readily available because, data sharing requirement at the request of the participants. Requests to access the datasets should be directed to wchinyamurindi@ufh.ac.za.

## Ethics statement

The studies involving human participants were reviewed and approved by University of Fort Hare Institutional Faculty Research Ethics Committee. The ethics committee waived the requirement of written informed consent for participation.

## Author contributions

The author confirms being the sole contributor of this work and has approved it for publication.

## Conflict of interest

The author declares that the research was conducted in the absence of any commercial or financial relationships that could be construed as a potential conflict of interest.

## Publisher's note

All claims expressed in this article are solely those of the authors and do not necessarily represent those of their affiliated organizations, or those of the publisher, the editors and the reviewers. Any product that may be evaluated in this article, or claim that may be made by its manufacturer, is not guaranteed or endorsed by the publisher.

## Appendix 1

### Interview questions

- Can you tell me a bit about yourself [Probe: years of work experience, position, and managerial duties].
- Based on your experience, what have been the positives of remote working?
- Based on your experience, what have been the negatives of remote working?
- Overall, do you think working remotely will suit the South African public service context? [Probe: what must be done to allow for this?].
